# Sampling pollen beetle (*Brassicogethes aeneus*) pressure in oilseed rape: which method is best?

**DOI:** 10.1002/ps.6310

**Published:** 2021-02-26

**Authors:** Gaëtan Seimandi‐Corda, Todd Jenkins, Samantha M Cook

**Affiliations:** ^1^ Biointeractions and Crop Protection Department Rothamsted Research Harpenden UK

**Keywords:** *Meligethes aeneus*, decision support, podless stalk, feeding damage, oviposition damage, control threshold, monitoring

## Abstract

**BACKGROUND:**

The pollen beetle (*Brassicogethes aeneus*) is the most abundant pest of oilseed rape in spring and is potentially one of the most damaging. Adults feed on the pollen within closed flower buds and the damage leads to bud abscission, resulting in podless stalks and yield reduction. Several methods are currently used to monitor the pressure of this insect, such as counting the numbers of adults on the plants, quantifying the number of buds damaged by the insect before flowering or counting the number of podless stalks before harvest. We conducted experiments to evaluate the robustness of these sampling methods and compared their results. We also describe how pollen beetles damage the plants to understand the limitations of methods based on damage estimation.

**RESULTS:**

Methods based on adult abundance lack robustness. We observed that most of the damage to buds is caused by pollen beetles feeding on small buds (< 3 mm), and that this damage can be quantified later in the season, indicating that methods based on the count of podless stalks are robust. Different methods gave consistent results and quantification of the pressure on the primary raceme can be a good proxy for pressure on the whole plant.

**CONCLUSIONS:**

Standardised methods for assessment of pollen beetle pressure will enable comparison of pest management strategies between different studies and facilitate the development of alternative control strategies for this pest.

© 2021 Rothamsted Research. *Pest Management Science* published by John Wiley & Sons Ltd on behalf of Society of Chemical Industry.

## Graphical abstract

Podless stalks count on oilseed rape is a reliable estimator of pollen beetle pressure after flowering. Count of damaged buds is a better estimator of pest pressure before flowering than adult abundance and could be favoured to develop a better control threshold.

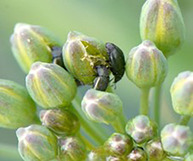

## 1 INTRODUCTION

Oilseed rape (OSR; *Brassica napus*) is the world's second most important oilseed crop, after soybean,^1^ and the most cultivated oilseed crop in Europe with 5.58 mol L^–1^ ha grown in 2019.^2^ In Europe, winter‐sown OSR predominates over spring‐sown systems and is usually sown between July and September and harvested the following July–August. During this long cultivation period multiple biotic stresses, such as pathogens, molluscs and insects, can potentially damage the plant. Consequently, pesticides, especially insecticides, are frequent inputs used by farmers to successfully grow this crop and attain maximum yield.^3^ However, since the ban on neonicotinoid use in Europe (EU Regulation No. 485/2013) and the development of resistance to pyrethroid insecticides in several OSR pest species^4^, ^5^, ^6^, ^7^, ^8^ insect control in OSR is becoming increasingly problematic.^9^, ^10^ This situation has led to an increasing need for new integrated pest management strategies.^11^ To develop these strategies we need to better understand some aspects of the ecology of OSR pests, but there is also a need to adopt effective, standardised sampling methods so that efficacy can be more easily compared between studies.

The pollen beetle, *Brassicogethes aeneus* (formerly *Meligethes aeneus*) Fabricius (Coleoptera: Nitidulidae), is one of the major pests of OSR in Europe.^12^ This insect can cause serious yield reduction in both winter and spring OSR.^13^ In Germany loss of control due to pyrethroid resistance in 2006 led to the damage of 200 000 ha and the complete loss of 30 000 ha, valued at approximately €25 million.^14^ Subsequently, a recent study placed the pollen beetle as the main pest of OSR in Sweden.^15^ Assessment of the relative importance of pests is not available for other European countries; however, pollen beetles are the main target for spring insecticide sprays and treatment is common in most European countries.^16^ Adult pollen beetles emerge from hibernation sites in early spring and migrate by flight to fields of OSR when the temperature reaches about 12°C .^17^ Adults are generalist pollinivores,^18^ but once they migrate to OSR for reproduction they eat pollen within the developing flower buds or from open flowers if available.^19^ They oviposit in the floral buds of OSR (and other Brassicaceae) where their larvae develop.^20^, ^21^ Pollen beetle feeding may completely destroy the floral bud, or injure the ovary, leading to bud abortion and failure of development of pods and seeds. Oviposition or larval feeding can also cause bud loss, but it seems that most bud loss is due to adult feeding attacks.^19^ Several studies point to the fact that small buds suffer most from adult feeding damage, whereas medium size buds appear to be used more often for oviposition.^17^, ^22^, ^23^, ^24^ However, these studies are mostly laboratory‐based bioassays and bud use has not been quantified in the field. More detailed field observation is needed to describe how adult pollen beetles use OSR buds, and the relationship between feeding and oviposition damage with bud size.

The importance of pollen beetle on OSR productivity has led to a considerable amount of work by the research community, and the publication of a large body of literature dealing with this insect. A systematic literature search reveals that more than 273 studies have been published on pollen beetles. This can be compared with 81 published studies on the cabbage stem flea beetle (*Psylliodes chrysocephala*), 77 for the cabbage seed weevil (*Ceutorhynchus obstrictus*), 47 for the brassica pod midge (*Dasineura brassicae*) and 42 for the cabbage stem weevil (*C. pallidactylus*) as the other major pests commonly associated with OSR (Web of Science Core Collection, database accessed 8 July 2020). When studying this literature, it was surprising to discover that the authors use diverse sampling techniques to estimate pollen beetle pressure.^25^ In some cases, studies focused on the number of adult insects trapped using yellow water traps^26^, ^27^, ^28^, ^29^ or sticky traps.^30^, ^31^ Others focused on the number of adult insects sampled by beating plants,^28^, ^32^, ^33^, ^34^, ^35^, ^36^, ^37^, ^38^, ^39^ sweep netting^29^, ^32^, ^40^ or vacuum suction sampling.^41^ Some studies also reported on the damage caused by the pollen beetle and directly assessed the number of buds damaged before flowering^35^, ^42^; or more commonly, the number of podless stalks after flowering.^19^, ^32^, ^43^

**FIGURE 1:**
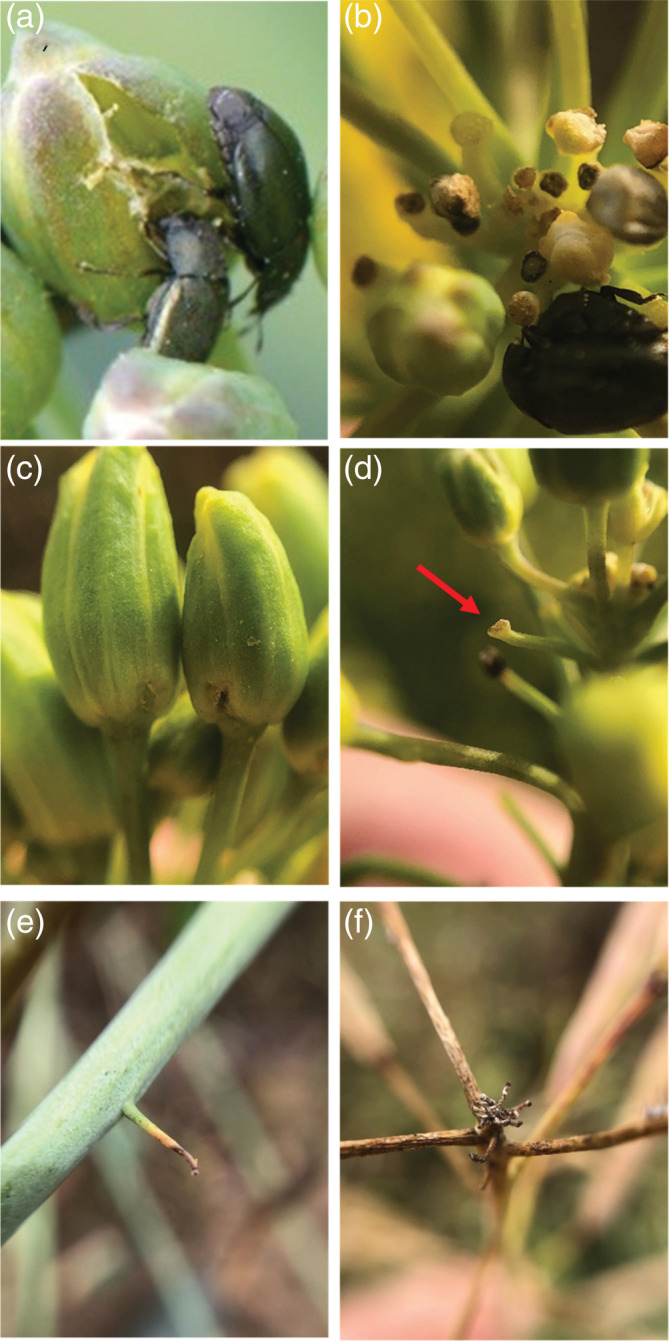
Pollen beetle damage to oilseed rape (OSR) plants: (a) pollen beetles feeding on a large bud and leaving a large, irregular‐shaped hole; (b) pollen beetle feeding on a small bud at the terminal point of an OSR raceme, neighbouring small buds showing pollen beetle feeding damage; (c) typical regular, oval‐shaped pollen beetle oviposition holes at the base of OSR buds; (d) arrow pointing to a pedicle without a bud or pod (‘podless stalk’) on the terminal raceme of an OSR plant with buds; (e) podless stalks on a stem before plant desiccation; (f) group of podless stalks observed at the top of the raceme not resulting from pollen beetle attacks.

Methods dealing with insect abundance are commonly used to monitor pollen beetle migration to the field, but they have also been used to estimate insect pressure as they can be easily linked to control thresholds. However, the output of such methods can be variable because adult pollen beetles are highly mobile.^44^, ^45^ The climatic conditions, especially temperature at the time of assessment, can have a strong impact on the number of insects found, which can lead to a biased estimation of the pressure.^46^ Assessment of direct feeding damage is usually made just before flowering, when plants are the most susceptible to the insect, by direct observation of the characteristic damage caused by the pollen beetle (i.e. counting the proportion of buds with feeding holes; Figure 1a,b). However, pollen beetle feeding damage often leads to bud abscission and failure of buds to produce pods. In this case, bud abscission leaves a small pedicle without a bud or a pod, called a ‘blind stalk’ or a ‘podless stalk’ (Figure 1d,e), on the main stem or secondary branches and these are easy to identify once all the pods have developed.^19^, ^47^, ^48^ The term ‘podless stalk’ is used throughout this study to refer to this type of damage. Several authors have pointed out that podless stalks can be caused by physiological limitations or by abiotic factors, including frost and drought, and occur in pest‐free environments^19^, ^49^; leading to an overestimation of the pest pressure. However, short pedicles left by attacks on smaller buds may not be visible at the end of plant development and this could lead to an underestimation of the pressure. Furthermore, even within a particular assessment technique, pollen beetle sampling methods are often not carried out using standardised means. Some studies recorded data over whole plants whereas others focus only on one raceme (usually the main or primary raceme). Evidence that data from the main raceme are correlated with that collected from the whole plant is scarce (but see Pouzet and Ballanger^50^). In order to develop efficient integrated pest management strategies, testing and comparing different sampling techniques are essential to determine accurate, standardised sampling procedures that can be reproduced, enabling results to be easily compared between studies.

This study had four aims: (i) to improve the accuracy of studies that count the number of damaged buds, we sampled OSR plants in the field to quantify pollen beetle damage caused by feeding or oviposition and the relationship with bud size; (ii) to explore the accuracy of population estimates based on the number of adult beetles per plant, we counted the number of pollen beetles on the same OSR plants on different occasions to check if this method produced stable and reliable results; (iii) to understand variation between different sampling techniques and methods commonly used, we also assessed pollen beetle pressure using different sampling techniques (counts of adult insects, damage at bud stage and counts of podless stalks after flowering) and compared the data from the main raceme only with that from the whole plant; and (iv) to explore the potential underestimation of pollen beetle damage to small buds using the podless stalk method, we conducted an experiment under controlled conditions to check if abortion of small buds whose pedicle is extremely small leads to identifiable podless stalks. These experiments draw together data on pollen beetle behaviour and damage effects to help determine which sampling method is most appropriate to monitor pollen beetle pressure in the field.

## 2 MATERIALS AND METHODS

### 2.1 Experimental site

All experiments were conducted in spring 2019 on Rothamsted Farm, U.K. Eight fields of OSR were used in this work; field size ranged between 0.95 and 5.9 ha with a minimum distance of 100 m between them. They were drilled with winter OSR (cv. Campus, PT240CL or Barbados) in late August 2018 and received standard agricultural management for the region (Table S1). Spring insecticide (240 g L^−1^ thiacloprid, Biscaya, Bayer Crop Science) was applied in mid‐April to the crop, except in two fields (Highfield and New Zealand) (Table S1).

### 2.2 Description of pollen beetle damage

Ten plants at the yellow bud growth stage, just before flowering (growth stage BBCH 57 according to Lancashire *et al*.^51^), were randomly selected from a field of OSR at least 25 m from the field edge (Highfield field, 3 March 2019) and brought to the laboratory for detailed inspection. All racemes on these plants were carefully checked to identify buds with pollen beetle damage. Damaged buds were categorised into three groups: buds used for feeding, that is those with large, irregular holes within the central area or bite marks (Figure 1a,b); buds used for oviposition, that is when a neat, oval hole was observed at the base of the bud (Figure 1c); and non‐identified damage when buds aborted leaving only a pedicle (Figure 1d). In this last situation, it was not possible to determine if the bud loss was due to feeding or oviposition damage caused by the pollen beetle or due to other factors. When buds with damage were identified, they were measured under a microscope using a ruler (0.1 mm precision) and assigned to one of three size categories: less than 3 mm, 3 — 5 mm and greater than 5 mm, defined according to previous observations.^22^, ^24^, ^52^

Differences in the number of damaged buds occurring per damage category (feeding, oviposition and non‐identified podless stalk) were tested using a Wald chi‐square test applied on a linear mixed model (LMM) including the individual plant as a random factor and pairwise comparisons of estimated marginal means (EMM), which were used to compare the three responses. The same procedure was used to compare the numbers of buds used for feeding and oviposition according to the categories of bud size. We also checked for correlation between the number of buds used for oviposition and for feeding per plant using Pearson's test. All statistical tests were performed using R software 3.6.1^53^ and the R‐packages lme4,^54^ car,^55^ emmeans,^56^ and multcomp.^57^

### 2.3 Accuracy and relationship between different estimators of pollen beetle infestation

#### 2.3.1 Variability of pollen beetle abundance over time and assessments from the main raceme versus whole plant

A set of 30 plants was sampled on two consecutive days from a patch of OSR crop with homogeneous growth just before flowering (BBCH 57) located approximately 25 m from the field edge (Highfield). On the first day the number of adult pollen beetles and the number of damaged buds (buds with feeding damage and podless stalks) were carefully counted on the main raceme and the whole plant by visual inspection only, so as not to disturb the beetles. Plants were labelled with a plastic tag for identification the following day. A second count of beetles and damaged buds was conducted the following day at the same time on the same plants to assess the temporal variability of the number of beetles. These observations were carried out between 14:00 h and 16:00 h on two occasions on different plants (21–22 March and 27–28 March 2019). Weather conditions were also recorded. The variability of the measures of pollen beetle abundance were tested by comparing the number of beetles counted on the two consecutive days for the two sampling occasions using Pearson's correlations. All statistical tests were performed using R software 3.6.1.^53^

#### 2.3.2 Correlation between different sampling methods and assessments from the main raceme versus whole plant

To test the correlation between adult pollen beetle abundance recorded on the plants and the number of damaged buds we used the data collected from the previous sample (collected on 27 March 2019 from plants just before flowering, i.e. BBCH 57). These data were supplemented with data collected on another set of 30 plants sampled on the same date (27 March 2019) using the same methods but from a less‐developed patch of plants at BBCH 55 (with individual flower buds on the main inflorescence visible but still closed) to ensure that the results were not growth stage dependent. Correlations between the abundance of adults recorded on the plants and the number of damaged buds were tested using Pearson's correlations for each growth stage and between the whole plant and the main inflorescence.

To estimate the relationship between the number of damaged buds during the pre‐flowering phase and the number of podless stalks before harvest, 25 plants were selected from each of eight fields of OSR before flowering (on 10 April 2019; crop growth stage was between BBCH 57 and BBCH 60, i.e. between the yellow bud growth stage and the first open flower). The number of damaged buds was then counted on the main raceme. In each field, these plants were chosen randomly along a transect 30 m long which was parallel to and 5 m from the field edge. A plastic tag was placed on these plants to identify them later in the season. Just before harvest, when pods were ripened (BBCH 97, 9–10 July 2019), the main racemes of these plants were collected. Six whole plants were also cut at the base and taken to compare the damage observed on the main inflorescence and that on the plant as a whole. These plants were stored for a maximum of 1 month in cool, dry conditions and all the inflorescences were checked for podless stalks. On each inflorescence, the number of podless stalks due to pollen beetles (Figure 1e), the number of pods and the number of podless stalks not related to pollen beetles (Figure S1) were counted. We considered podless stalks with a size equivalent to the stalks bearing fully developed pods as not related to pollen beetle attack. These could be due to post‐sampling loss or to loss of the pod following infestation by brassica pod midge larvae (*Dasineura brassicae*; pers obs). Floral buds at the tip of the inflorescence usually fail to produce pods (Figure 1f) ^40^, ^41^ and therefore the group of podless stalks normally found at the top of the raceme were not counted. Data on the number of damaged buds and podless stalks counted on the main racemes from the different fields were used to test the correlation between these two measurements using Pearson's correlation. Correlations were also tested individually for each field and their *P*‐values were corrected by the false discovery rate.^58^

To test for differences in the numbers of damaged buds and podless stalks between fields, two linear regressions were carried out. The first linear regression explained the number of damaged buds according to the field where the sampling was done and the growth stage at sampling (BBCH 55 or 57). The second explained the number of podless stalks according to field. F‐tests were used to test the effect of the cofactors on the damage level and pairwise comparisons of EMM were used to test differences between fields. The differences in the numbers of damaged buds and podless stalks were tested using Wald chi square tests applied on a LMM explaining the damage level by field, the type of measurement and the interaction of the two factors fitted as fixed effects. Plant identity was included as a random factor. Pairwise comparisons EMM were used to test differences between fields. All assessments were undertaken before insecticide spray applications (Table S1) except the count of podless stalks. To determine whether or not insecticide treatments affected the number of podless stalks observed on the primary raceme a Wald chi square test was applied on a LMM including the treatment of the field (sprayed or not) as fixed factor and the field as a random factor. All statistical tests were performed using R software 3.6.1^53^ and the R‐packages lme4,^54^ car,^55^ emmeans,^56^ and multcomp.^57^

### 2.4 Validation of the podless stalk method in controlled conditions

To test if pollen beetle feeding on small buds leads to visible podless stalks, two experiments were conducted in a glasshouse. OSR plants (cv. Apex) were grown in individual propagation plugs for approximately 2 weeks within the glasshouse (16:8 h light/dark photoperiod, 21°C daytime and 18°C night) until they reached the three‐ to four‐leaf stage. They were then vernalised (8:16 h light/dark photoperiod, 6°C) for 8 weeks. Plants were subsequently transplanted into 2‐L pots and grown in an unheated glasshouse.

In experiment I, buds and their pedicles were detached from the main raceme of ten plants at growth stage BBCH 59 (just before the first flowers appear) (366 buds in total, ranging from 0.4 to 8.2 mm in size). Bud and pedicle lengths were measured with a ruler (0.1 mm precision) under a microscope. These data were used to model the relationship between the bud size and the pedicle length. The significance of this relationship was tested using an F‐test applied on a LMM including the plant as a random factor.

In experiment II, one bud was removed from the main raceme of individual plants (growth stage BBCH 57) using sharp forceps to simulate bud abscission. Buds of two sizes were removed: a large bud (mean = 5.8 mm, min = 4.5 mm, max = 7.8 mm) or a small bud (mean = 1.5 mm, min = 0.6 mm, max = 2.4 mm). Buds were removed from 25 plants for each size (large and small buds) and measured with a ruler (0.1 mm precision) under a microscope. Because of the small size of the pedicles attached to small buds it was not possible to mark them. Plants were then kept in randomised positions in an unheated glasshouse. The main raceme was cut when it reached maturity (BBCH 67) and the podless stalks left by bud removal were checked. When it was possible to observe these pedicles they were cut from the stem with a scalpel and measured with a ruler (0.1 mm precision as above). Plants were potted on two occasions and bud removal was carried out on the first set of plants between 13 and 20 May 2019 (40 plants) and on the second group on 10 June 2019 (ten plants). The main racemes were cut on 27 June 2019 and 1 July 2019 for the first and second set, respectively. A binomial one‐tailed test was used to test if the proportion of plants with a podless stalk observed at the end of the experiment was lower when a small bud was removed than when a large bud was removed. To understand whether pedicles grow after bud removal, the model built for experiment I was used to estimate the length of the pedicle of the buds that were removed from the raceme. It was then possible to compare the estimate length of the pedicle at the time of bud removal and the observed length at the end of the experiment to estimate the increase in pedicle length. An F‐test was done to compare pedicle length and increase in pedicle length when a small or a large bud was removed. All statistical tests were performed using R software 3.6.1^53^ and the R‐packages lme4,^54^ car.^55^

## 3 RESULTS

### 3.1 Description of pollen beetle damage

**FIGURE 2:**
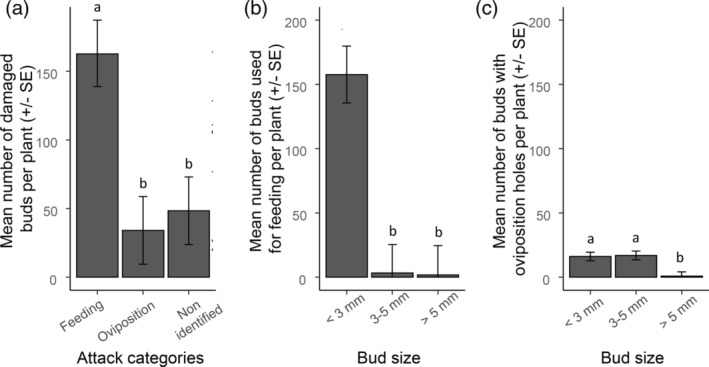
Estimated marginal means (± SE) number of damaged buds per oilseed rape plant from a sample of ten plants collected at the yellow bud stage (just before flowering) with: (a) evidence of pollen beetle damage caused by feeding or oviposition damage or pedicles with no buds resulting from non‐identified attacks (podless stalks); (b) feeding damage according to bud size; and (c) oviposition damage according to bud size. Different letters indicate significant differences according to the linear mixed model analysis.

Before OSR flowering we found differences between the numbers of buds used for feeding, oviposition and bud abscission due to unidentified factors on the plant (*χ* ^2^_2_ = 24.62, *P* < 0.001) (Figure 2a). Most damage was caused by pollen beetle feeding, whereas buds used for oviposition and podless stalks were less numerous. We also found differences between the number of buds used for feeding according to the different size classes (*χ* ^2^_2_ = 33.06, *P* < 0.001) (Figure 2b). The vast majority of the buds used for feeding were small, with medium and large buds displaying feeding damage infrequently. Differences were also found in the numbers of buds used for oviposition between the different class sizes (*χ* ^2^_2_ = 22.28, *P* < 0.001) (Figure 2c). Oviposition holes were equally distributed between small and medium size buds and were less frequent on large buds. We observed a strong positive correlation between the number of buds used for feeding and the number of buds used for oviposition (*r* = 0.91, *df* = 8, *P* < 0.001).

### 3.2 Accuracy and relationship between different estimators of pollen beetle infestation

#### 3.2.1 Pollen beetle abundance is variable over time

**FIGURE 3:**
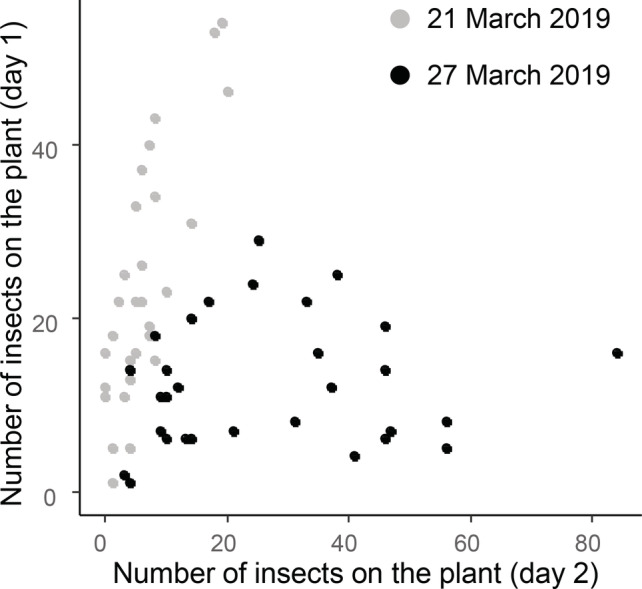
Relationships between the numbers of pollen beetles counted on whole oilseed rape plants (BBCH 57) in the field (day 1) and the numbers found on the same plants the next day (day 2) on two occasions: 21–22 March 2019 (shown in grey) and 27–28 March 2019 (shown in black).

The numbers of pollen beetles were counted on the same plants on two consecutive days on two sampling occasions. A strong positive correlation was observed between the number of beetles counted on the first and second day for the first sampling occasion on 21–22 March 2019 (*r* = 0.80, *df* = 28, *P* < 0.001), but the correlation was not significant for the second sampling occasion on 27–28 March 2019 (*r* = 0.10, *df* = 28, *P*= 0.6) (Figure 3). Similar results were found if we consider data from the main raceme only (21–22 March 2019: *r* = 0.59, *df* = 28*, P* < 0.001; 27–28 March 2019: *r* = −0.16, *P* = 0.4). These data demonstrate that measures of adult abundance can be variable from day to day.

#### 3.2.2 Correlation between several sampling methods

**FIGURE 4:**
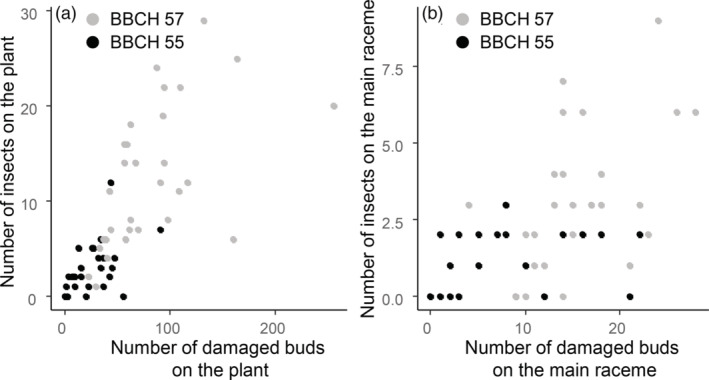
(a) Number of adult pollen beetles per oilseed rape plant and correlation with the number of damaged buds per plant. (b) Number pollen beetles and the correlation with the number of damaged buds on the main raceme only. Plants (*n* = 56) collected in the field between 21 and 27 March 2019. Plants at BBCH 55 (with individual flower buds visible but still closed) are indicated in black and plants with individual flower buds (secondary inflorescences) visible but still closed (BBCH 57) are indicated in grey.

**FIGURE 5:**
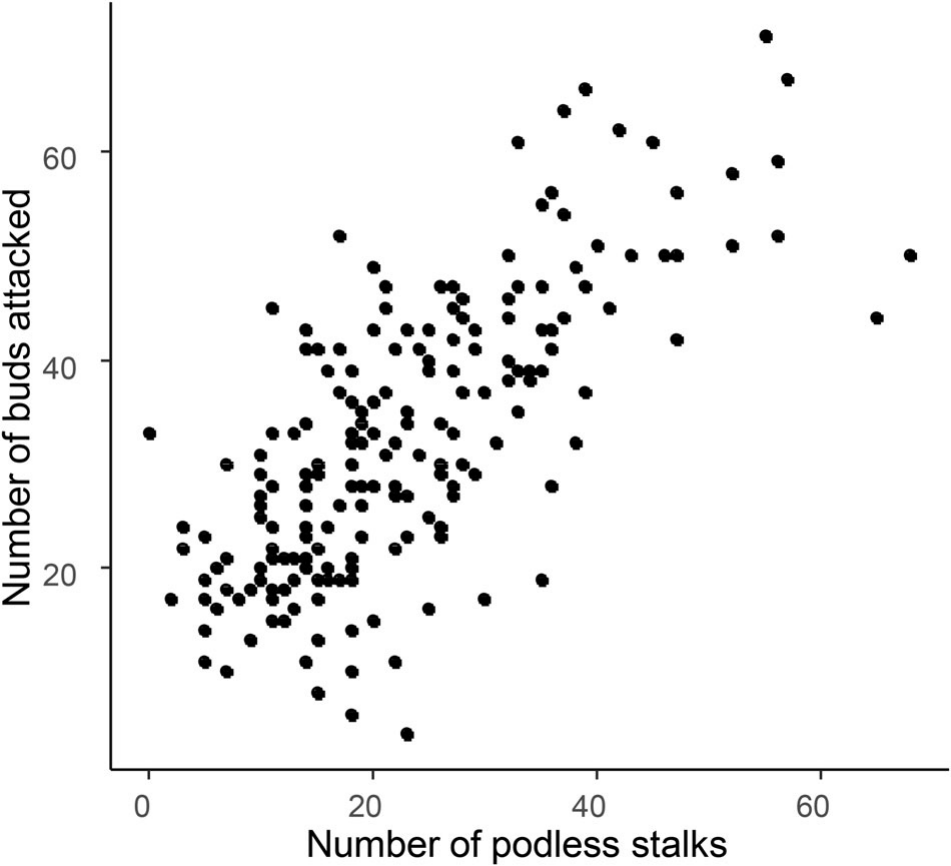
Number of oilseed rape buds damaged by pollen beetles on the main raceme and correlation with the number of podless stalks on the main raceme before harvest.

Correlations between the number of adult pollen beetles and the number of damaged buds were apparent for both plant growth stages sampled (BBCH 55: *r* = 0.49, *df* = 24, *P* = 0.012; BBCH 57: *r* = 0.56, *df* = 28, *P* = 0.001) (Figure 4a). However, if only the data collected on the main raceme are considered, no correlation was found between the numbers of insects and the damaged buds observed at BBCH 55 (*r* = 0.38, *df* = 24, *P* = 0.057) although a significant correlation was found on plants sampled at BBCH 57 (*r* = 0.43, *df* = 28, *P* = 0.017) (Figure 4b). A significant correlation was also found between the numbers of damaged buds on the main racemes collected in different fields and the numbers of podless stalks on these inflorescences before harvest (*r* = 0.72, *df* = 195, *P* < 0.001) (Figure 5). Correlations of these data for individual fields were significant for only four fields but were always positive (Table S2). Significant differences in the damage level between fields and growth stages were observed before flowering (field: *F*_7,188_ = 28.34, *P* < 0.001; growth stage: *F*_1,188_ = 5.41, *P* = 0.021). Significant differences between the number of podless stalks between fields was also observed (*F*_7,188_ = 15.01, *P* < 0.001). Significant differences between the number of damaged buds and the number of podless stalks before harvest were found for each field except one (Table 1). There was no effect of insecticide spray on the number of podless stalks per primary raceme (*χ* ^2^_1_ = 0.003, *P* = 0.995).

**TABLE 1:** Estimated marginal means (EEM) ± SE number of pollen beetle damaged buds and podless stalks in oilseed rape crops in eight different fields on Rothamsted farm (UK) and results of Wald chi‐square tests for the differences in the number of pollen beetle attacks on oilseed rape buds recorded when plants were sampled at the bud stage (numbers of damaged buds) and before harvest (numbers of podless stalks)

Field	EMM number of damaged buds (± SE)	EMM number of podless stalks (± SE)	*χ* ^2^_1_	*P*‐value	
Delafield	21.061 (± 1.928)	15.200 (± 2.056)	8.13	0.035	*
Furzefield	20.851 (± 1.913)	17.480 (± 2.056)	3.64	0.450	Ns
Great Knott	30.074 (± 1.968)	23.333 (± 2.094)	11.49	0.006	**
Highfield	27.318 (± 1.947)	14.958 (± 2.097)	64.68	0.000	***
Long Hoos	36.896 (± 1.902)	23.600 (± 2.056)	63.89	0.000	***
New Zealand	45.078 (± 1.896)	30.160 (± 2.056)	67.02	0.000	***
Osier	30.643 (± 1.904)	19.720 (± 2.056)	85.87	0.000	***
Webbs	48.099 (± 1.968)	38.500 (± 2.097)	12.96	0.003	**

ns, *P* > 0.05; *P < 0.05; **P < 0.01; ***P < 0.001.

#### 3.2.3 Damage amount on the main raceme is correlated with that on the whole plant

**FIGURE 6:**
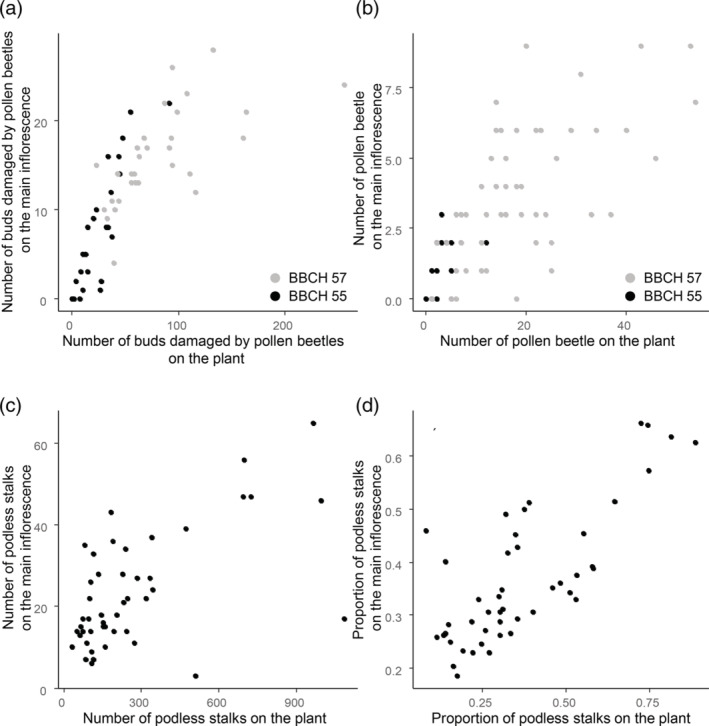
Relationships between measures of pollen beetle pressure on the whole plant with that on the main raceme only. (a) Numbers of oilseed rape buds damaged by the pollen beetle. Plants sampled 21–27 March 2019; plants with individual flower buds visible but still closed (BBCH 55) are indicated in black and plants with individual flower buds (secondary inflorescences) visible but still closed (BBCH 57) are indicated in grey. (b) Numbers of pollen beetle adults. Plants collected in the field between 21–27 March 2019; BBCH 55 (black), BBCH 57 (grey). (c) Numbers of podless stalks just before harvest; plants collected from OSR crops (BBCH 97) from eight different fields. (d) Proportion of podless stalks. Plants collected from oilseed rape crops just before harvest (BBCH 97) from eight different fields.

With the information collected from the different fields it was possible to estimate the correlation between the data collected on the main raceme and the whole plant for the abundance of adult pollen beetles, the numbers of damaged buds and the numbers of podless stalks at growth stages BBCH 55 and BBCH 57. Strong and significant correlations between measures on the main raceme and the whole plant were recorded for all the measurements (Figure 6). This was true for the number of buds damaged (BBCH 55: *r* = 0.88, *df* = 24, *P* < 0.001; BBCH 57: *r* = 0.66, *df* = 58, *P* < 0.001), the number of adult pollen beetles (BBCH 55: *r* = 0.55, *df* = 24, *P* = 0.003; BBCH 57: *r* = 0.63, *df* = 58, *P* = 0.001), the number of podless stalks (*r* = 0.61, *df* = 44, *P* < 0.001) and the proportion of podless stalks (*r* = 0.77, *df* = 44, *P* < 0.001).

### 3.3 Pollen beetle damage leads to identifiable podless stalks

In glasshouse experiment I we measured the length of buds removed and the pedicle length on OSR inflorescences and observed that this relationship is best modelled using a second‐degree polynomial regression (approximated *R* ^2^ adjusted from LMM = 0.93, *F*_2,271_ = 1848.2, *P* < 0.001) (Figure S2). This allowed us to build a predictive model to infer the original pedicle length of buds removed during experiment II. Values obtained with the predictive model are highly correlated with the observed values (*r* = 0.97, *df* = 272, *P* < 0.001).

In glasshouse experiment II we were able to identify the pedicle of removed buds in the majority of cases, even when those removed were very small; 20 of 25 pedicles from small buds and 21 of 25 pedicles of large buds removed were visible at the end of the experiment. The pedicles resulting from the removal of small buds were not more difficult to identify than those following the removal of large buds (binomial one‐tailed test, *P* = 0.370). Some pedicles were not found because additional buds aborted due to other factors (e.g. lack of pollination, physiological abortion, thrips feeding attacks) on the same inflorescence and made their identification uncertain. Using the model developed in experiment I and the data on pedicle length at the end of the experiment II we estimated the growth of the pedicles to test if the length of the pedicle at the end of the development period is affected by the length of the pedicle when the bud aborts. Pedicles of abscised small buds grow less than those of large buds (*F*_1,45_ = 31.73, *P* < 0.001) leading to pedicles of different sizes (*F*_1,45_ = 217.81, *P* < 0.001) (Figure S3). By growing, pedicles of small buds become more visible and can be identified later as a podless stalk.

## 4 DISCUSSION

Our observations show that before flowering, feeding activity is the main cause of damage by pollen beetles in winter OSR, with at least 64% of buds damaged by feeding compared with 14% damaged by oviposition. This supports previous studies^20^ and suggests that feeding damage is the main contributor to yield loss by pollen beetle. Our observations also indicate that feeding was focused on small buds (96% in our experiment). This finding has previously been reported in laboratory and field experiments^17^, ^24^ but is quantified here for the first time.

We aimed to identify an efficient sampling method to estimate pollen beetle pressure. We found that sampling just the main raceme is an accurate estimator of that on the whole plant. We discovered a good correlation between the results collected on whole plants and on the main inflorescence for all the different sampling methods traditionally used (counts of adult insects per plant, direct bud damage before flowering and counts of podless stalks before harvest). Sampling the whole plant can be extremely time‐consuming, especially when assessing the numbers of podless stalks, which reached up to 1085 per plant in our experiments, so sampling just the main raceme can save time while not impacting on accuracy.

Comparison of adult pollen beetle abundance data collected on different dates from the same plants shows that this method to determine pollen beetle pressure can lack robustness. With a delay of only 24 h between measurements the results collected between days 1 and 2 differed significantly on one of the two sampling occasions and suggests a lack of reliability with this method. This may not be surprising as pollen beetles are good dispersers.^44^, ^45^ They are highly mobile and can rapidly move from one plant to another or perhaps even to another field to find more preferred resources. Differences in time of day, climatic conditions during assessment, plant growth stage and plant position in the field can also bias the estimation.^31^, ^46^ Sampling methods based on adult beetle abundance are certainly suitable (and required) for studies focusing on their population dynamics, or spatial and temporal aspects of crop colonisation. However, abundance, especially using a single estimation during the season, cannot be a reliable estimator of the pressure of this insect. Previous studies also found relatively low correlation between the abundance of adults recorded in the field and the damage level observed on the plants.^15^, ^19^, ^41^ Furthermore, Ferguson *et al*.^17^ found damage was influenced by temperature, so several pollen beetles at low temperatures would cause less damage than fewer beetles at higher temperatures. Most of the spray thresholds used in Europe for this pest are based on adult abundance per plant^12^, ^16^, ^59^ so our findings have important implications for the accuracy of threshold determination using this method. Counting the number of adult beetles per plant can be done relatively easily by farmers but our results suggest the estimation can be highly inaccurate. A threshold based on a direct estimation of damage, that is the number of damaged buds on the main raceme would be more precise. Spray thresholds based on damage levels already exist for other insect pests of OSR, such as the cabbage stem flea beetle (*Psylliodes chrysocephala*).^12^ Among the different methods tested, the number of damaged buds is the most precise estimation of pollen beetle pressure and can be done when attacks are still occurring, unlike the podless stalk method. Counting damaged buds could be used to more accurately monitor how pollen beetles are impacting the crop and for determining when treatment is necessary. However, the numbers of damaged buds were affected by growth stage,^42^ so growth‐stage specific thresholds may be required. The method could become time‐consuming when large numbers of plants at more advanced growth stages (i.e. with more buds) need to be assessed. Assessment is traditionally done in the laboratory under a binocular microscope and fresh material cannot be stored for more than a few days, even in a refrigerator. However, this can be overcome by directly observing damage in the field with the use of a hand lens to facilitate field‐based assessments, as suggested in this study.

The count of podless stalks just before harvest is, by its nature, not a suitable measure of pollen beetle pressure for the purposes of determining when action thresholds have been breached at the bud stage, where immediate results are required to ascertain the need for treatment. Rather, this method can be used by researchers to estimate pollen beetle damage on a large number of plant samples, that is to determine differences between certain treatments.^32^, ^43^ Concerns about its reliability are common^19^, ^49^ but despite this, no comparison between this method and other measures has previously been carried out. Our experiments show that counts of podless stalks are highly correlated with the number of buds damaged by pollen beetles observed before flowering. This result implies that podless stalks are a reliable estimator of the pollen beetle pressure. One of the major criticisms of the podless stalk method is its capacity to overestimate pollen beetle attacks by confounding insect damage with podless stalks caused by plant physiological limitations or abiotic stress. Environmental conditions in 2019 (the year the experiment was done) were not extreme (no late frosts or drought in the spring), so did not lead to massive bud abortions and consequently did not influence the strong correlations we found. Different conditions may disrupt this correlation and further research is needed to investigate this. Contrary to what we expected, we found fewer podless stalks before harvest than damaged buds before flowering. This could be due to an overestimation of the bud loss by the counting of damaged buds that can still develop and produce pods after an attack, but most feeding damage occurs on small buds that cannot survive these attacks. A more reliable hypothesis could be that podless stalks tended to underestimate the level of damage in our experiment. This could be explained by the fact that we did not count the pedicles at the top of the inflorescence. These buds usually abort at the end of the flowering period of the plant but can also contain podless stalks caused by insects.^47^, ^48^ However, it was not possible to count them because the pedicles in this part of the inflorescence are very small and difficult to observe (Figure 1f). The experiment conducted under controlled conditions also showed that podless stalks can be observed even if very small buds are destroyed by the insect. This is possible because pedicles continue to grow after bud removal, making them clearly visible. These results show that the count of podless stalks can be a robust and appropriate method to estimate pollen beetle pressure on OSR, and the ultimate efficacy measure of control.

To conclude, we show that counting the abundance of adult pollen beetles on a plant is not a robust and reliable estimator of pollen beetle risk. Counts of damaged buds at bud stage are a more reliable indicator of risk of yield loss, and treatment thresholds based on this direct measure need to be developed. Counts of podless stalks on the main raceme are a reliable estimator of pollen beetle pressure. This method applied to the main raceme only can be used to quickly and accurately estimate pollen beetle pressure and is an ultimate measure of efficacy of different control measures. Adoption of standardised measures would facilitate comparison between studies and the development of new control approaches.

## Supporting information


**TABLE S1** Agronomical information on the different oilseed rape (OSR) crops sampled in eight different fields on Rothamsted farm (UK, 2019).
**TABLE S2** Results of the correlation tests made between the numbers of oilseed rape buds damaged by pollen beetles on the main inflorescence before flowering and the numbers of podless stalks on the main inflorescence before harvest in eight different crops in on Rothamsted farm (UK, 2019). ns, *P* > 0.05; *P < 0.05; **P < 0.01; ***P < 0.001.
**FIGURE S1** Part of an oilseed rape stem on a desiccated plant with (A) a large podless stalk with a thick tip indicating that a pod partially developed before falling, and (B) a small, thin podless stalk left by the abortion of a bud.
**FIGURE S2** Relationship between size (mm) of the pedicle and the floral buds on oilseed rape racemes. Data collected from buds from ten main inflorescences of plants reared in a glasshouse.
**FIGURE S3** Length (mm) of pedicles measured at the end of the plant growth phase (BBCH 67) for oilseed rape buds removed (light grey) according to the size of buds removed (large or small). Estimated increase in length of pedicles after bud removal (dark grey bars). Upper case letters indicate significant differences for the increase in length of the pedicles, lower case letters indicate significant differences for the length of pedicles measured.
