# Supplementary material for: Sampling pollen beetle (Brassicogethes aeneus) pressure in oilseed rape: which method is best?
Source: Pest Manag Sci. 2021 Feb 26;77(6):2785–94. doi: 10.1002/ps.6310 (PMC8248050; doi:10.1002/ps.6310)
Supplement: Supplementary file 1 — TABLE S1 Agronomical information on the different oilseed rape (OSR) crops sampled in eight different fields on Rothamsted farm (UK, 2019). TABLE S2 Results of the correlation tests made between the numbers of oilseed rape buds damaged by pollen beetles on the main inflorescence before flowering and the numbers of podless stalks on the main inflorescence before harvest in eight different crops in on Rothamsted farm (UK, 2019). ns, P > 0.05; *P < 0.05; **P < 0.01; ***P < 0.001. FIGURE S1 Part of an oilseed rape stem on a desiccated plant with (A) a large podless stalk with a thick tip indicating that a pod partially developed before falling, and (B) a small, thin podless stalk left by the abortion of a bud. FIGURE S2 Relationship between size (mm) of the pedicle and the floral buds on oilseed rape racemes. Data collected from buds from ten main inflorescences of plants reared in a glasshouse. FIGURE S3 Length (mm) of pedicles measured at the end of the plant growth phase (BBCH 67) for oilseed rape buds removed (light grey) according to the size of buds removed (large or small). Estimated increase in length of pedicles after bud removal (dark grey bars). Upper case letters indicate significant differences for the increase in length of the pedicles, lower case letters indicate significant differences for the length of pedicles measured. [file PS-77-2785-s001.docx]

**Supporting information:**

Table S1. Agronomical information on the different oilseed rape (OSR) crops sampled in eight different fields on Rothamsted farm (UK, 2019).

| **Field name** | **Field size (ha)** | **Previous crop** | **OSR genotype/breeder** | **Drilling date** | **Seed rate (s/m²)** | **Insecticides used Product/ rate** | **Application date** |
| --- | --- | --- | --- | --- | --- | --- | --- |
| Delafield | 2.49 | Winter wheat | Barbados (KWS) | 22/08/2018 | 70 | Biscaya (300 ml/ha) | 12/04/2019 |
| Furzefield | 0.95 | Winter OSR | PT240CL (Pioneer) | 24/08/2018 | 60 | Biscaya (300 ml/ha) | 12/04/2019 |
| Great Knott | 4.21 | Winter barley | PT240CL (Pioneer) | 24/08/2018 | 50 | Biscaya (300 ml/ha) | 12/04/2019 |
| Highfield | 4.1 | Winter OSR | Campus (KWS) | 23/08/2018 | 70 | No | NA |
| Long Hoos | 5.9 | Winter wheat | Campus (KWS) | 24/08/2018 | 70 | Biscaya (300 ml/ha) | 12/04/2019 |
| New Zealand | 3.88 | Winter wheat | Campus (KWS) | 24/08/2018 | 70 | No | NA |
| Osier | 5.83 | Winter barley | Campus (KWS) | 23/08/2018 | 70 | Biscaya (300 ml/ha) | 12/04/2019 |
| Webbs | 2.9 | Winter wheat | Campus (KWS) | 23/08/2018 | 70 | Biscaya (300 ml/ha) | 12/04/2019 |

Table S2. Results of the correlation tests made between the numbers of oilseed rape buds damaged by pollen beetles on the main inflorescence before flowering and the numbers of podless stalks on the main inflorescence before harvest in eight different crops in on Rothamsted farm (UK, 2019). ns = p > 0.05, * = p < 0.05, ** = p < 0.01, *** = p < 0.001.

| **Field** | ***r*** | ***df*** | ***p*** | |
| --- | --- | --- | --- | --- |
| Delafield | 0.41 | 23 | 0.317 |  |
| Furzefield | 0.51 | 23 | 0.073 |  |
| Great Knott | 0.47 | 22 | 0.172 |  |
| Highfield | 0.66 | 22 | 0.003 | ** |
| Long Hoos | 0.71 | 23 | 0.001 | ** |
| New Zealand | 0.75 | 23 | < 0.001 | *** |
| Osier | 0.76 | 23 | < 0.001 | *** |
| Webbs | 0.45 | 22 | 0.218 |  |


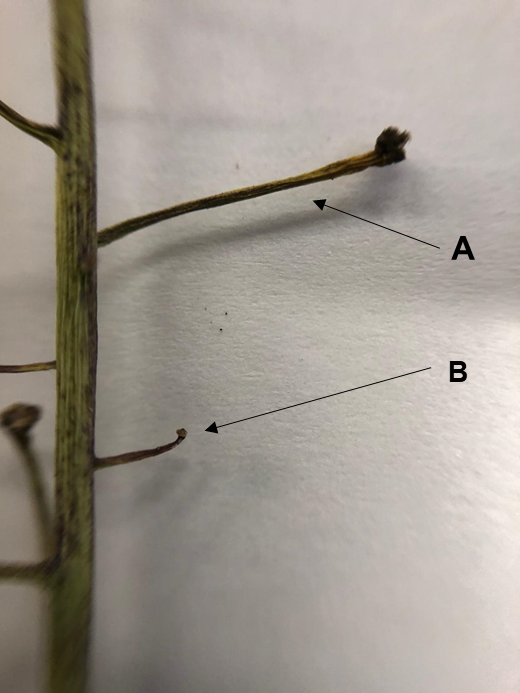


Figure S1. Part of an oilseed rape stem on a desiccated plant with A) a large podless stalk with a thick tip indicating that a pod partially developed before falling, and B) a small, thin podless stalk left by the abortion of a bud.

Figure S2. Relationship between size (mm) of the pedicle and the floral buds on oilseed rape racemes. Data collected from buds from ten main inflorescences of plants reared in a glasshouse.

Figure S3. Length (mm) of pedicles measured at the end of the plant growth phase (BBCH 67) for oilseed rape buds removed (light grey) according the size of buds removed (large or small). Estimated increase in length of pedicles after bud removal (dark grey bars). Upper-case letters indicate significant differences for the increase in length of the pedicles, lower case letters indicate significant differences for the length of pedicles measured.
